# Supplementary material for: The interactome of intact mitochondria by cross-linking mass spectrometry provides evidence for coexisting respiratory supercomplexes
Source: Mol Cell Proteomics. 2017 Dec 8;17(2):216–32. doi: 10.1074/mcp.RA117.000470 (PMC5795388; doi:10.1074/mcp.RA117.000470)
Supplement: Supplemental Data [file supp_RA117.000470_133922_0_supp_23978_fzffwf.zip › spectra_annotation/mito_spectra_annotation/428-1-6-1-8-1.pdf]

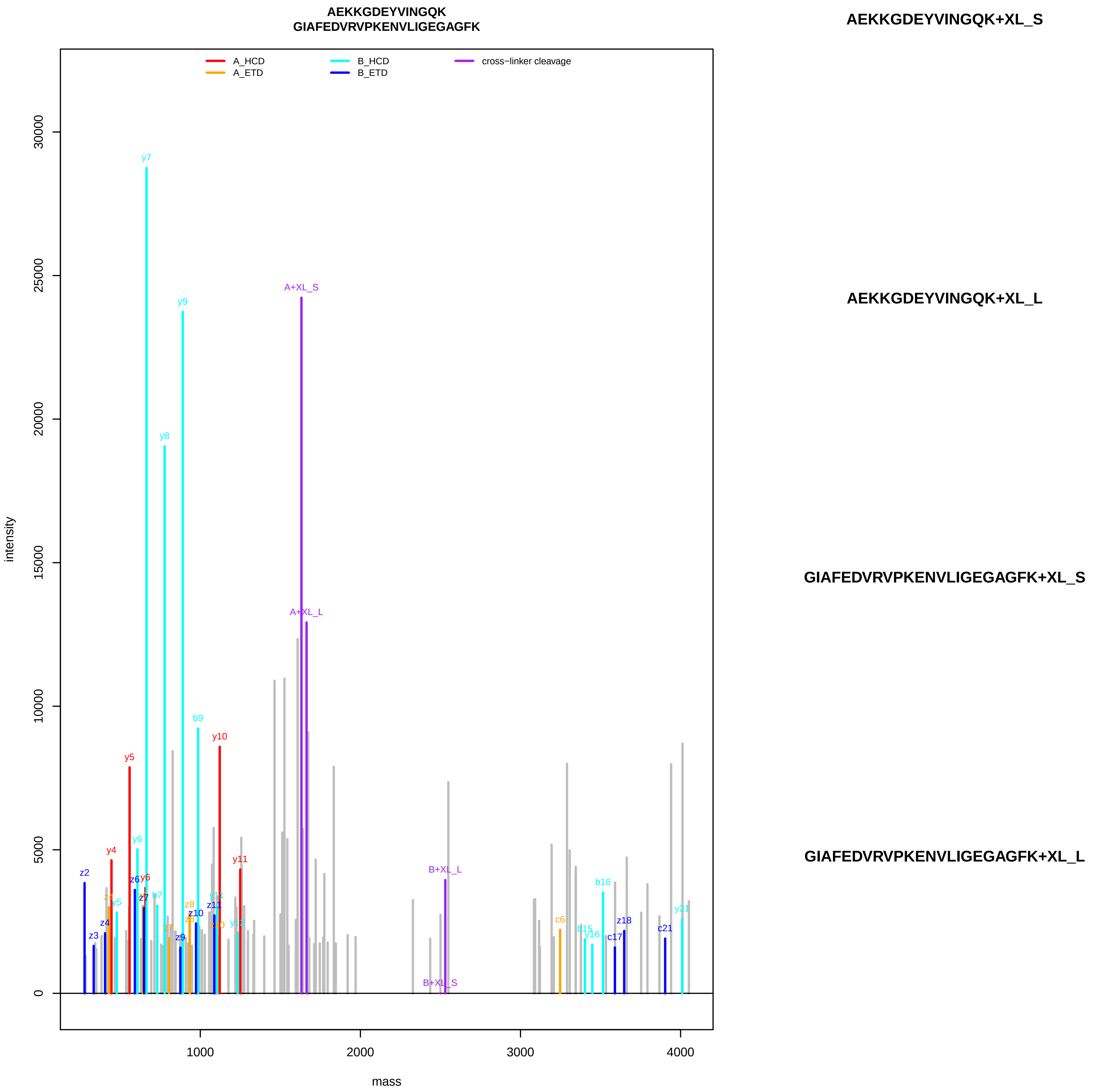
AEKKGDEYVINGQK
GIAFEDVRVPKENVLIGEGAGFK
A_HCD
A_ETD
B_HCD
B_ETD
cross−linker cleavage
intensity
mass
A+XL_S
A+XL_L
B+XL_L
B+XL_S
y7
y9
y8
b9
y10
y5
y11
y4
y6
z2
z3
z4
z6
z7
z8
z9
z10
z11
c6
b15
y16
b16
c17
z18
c21
y21
AEKKGDEYVINGQK+XL_S
AEKKGDEYVINGQK+XL_L
GIAFEDVRVPKENVLIGEGAGFK+XL_S
GIAFEDVRVPKENVLIGEGAGFK+XL_L
